# IGF-1 Gene Transfer to Human Synovial MSCs Promotes Their Chondrogenic Differentiation Potential without Induction of the Hypertrophic Phenotype

**DOI:** 10.1155/2017/5804147

**Published:** 2017-06-27

**Authors:** Yasutoshi Ikeda, Morito Sakaue, Ryota Chijimatsu, David A. Hart, Hidenori Otsubo, Kazunori Shimomura, Henning Madry, Tomoyuki Suzuki, Hideki Yoshikawa, Toshihiko Yamashita, Norimasa Nakamura

**Affiliations:** ^1^Department of Orthopaedic Surgery, School of Medicine, Sapporo Medical University, Sapporo, Japan; ^2^Department of Orthopaedic Surgery, Osaka University Graduate School of Medicine, Suita, Japan; ^3^McCaig Institute for Bone and Joint Health, University of Calgary, Calgary, AB, Canada; ^4^Department of Orthopedic Surgery, Saarland University Medical Center, Homburg, Saar, Germany; ^5^Global Center for Medical Engineering and Informatics, Osaka University, Suita, Japan; ^6^Institute for Medical Science in Sports, Osaka Health Science University, Osaka, Japan

## Abstract

Mesenchymal stem cell- (MSC-) based therapy is a promising treatment for cartilage. However, repair tissue in general fails to regenerate an original hyaline-like tissue. In this study, we focused on increasing the expression levels for insulin-like growth factor-1 (IGF-1) to improve repair tissue quality. The IGF-1 gene was introduced into human synovial MSCs with a lentiviral vector and examined the levels of gene expression and morphological status of MSCs under chondrogenic differentiation condition using pellet cultures. The size of the pellets derived from IGF-1-MSCs were significantly larger than those of the control group. The abundance of glycosaminoglycan (GAG) was also significantly higher in the IGF-1-MSC group. The histology of the IGF-1-induced pellets demonstrated similarities to hyaline cartilage without exhibiting features of a hypertrophic chondrocyte phenotype. Expression levels for the Col2A1 gene and protein were significantly higher in the IGF-1 pellets than in the control pellets, but expression levels for Col10, MMP-13, ALP, and Osterix were not higher. Thus, IGF-1 gene transfer to human synovial MSCs led to an improved chondrogenic differentiation capacity without the detectable induction of a hypertrophic or osteogenic phenotype.

## 1. Introduction

Articular cartilage (AC) has limited self-repair capabilities due in part to poor vascularity, no lymphatic system, and no innervation. In addition, AC is rich in a hyaline-like extracellular matrix abundant in type II collagen, glycosaminoglycan, and water, a composition which can interfere with rapid cellular migration and repopulation and thus negatively affect tissue repair processes. In order to compensate for such disadvantages, a variety of approaches have been investigated to improve cartilage healing [1–7]. Among them, stem cell therapy could be a promising option to facilitate regenerative repair. Mesenchymal stem cells (MSCs) have the capability to differentiate into a variety of connective tissue cells including bone, cartilage, tendon, muscle, and adipose tissue [8]. These MSCs may be readily isolated from many sources such as the bone marrow, skeletal muscle, synovial membranes, adipose tissue, and umbilical cord blood [9–16]. Specifically, MSCs isolated from synovial membrane may be well suited for cell-based therapies for cartilage repair because of the relative ease of their harvest and their strong capability for chondrogenic differentiation [14, 17, 18]. Recent implantation studies of synovial MSC (Syn-MSC) have shown successful repair of cartilage defects [18–21]. However, detailed observation revealed that in most cases, the repair tissues differed from normal hyaline cartilaginous tissue with contamination of some fibrocartilaginous or fibrous tissues [22]. Such findings suggest a need to further improve the quality of the repair tissue towards more complete regeneration.

One of the options to address the need for improvements could be biological manipulation of the differentiation capacity of MSC. Insulin-like growth factor (IGF-1) is known as one of the important growth factors that can regulate the chondrogenic potential of cells and chondrocyte status. Previous reports suggested that IGF-1 could play important roles in cartilage repair [23]. IGF-1 promotes the proliferation of MSCs [24] and also leads to increased production of aggrecan and type 2 collagen and thus contributes to maintaining the phenotype of chondrocytes [23, 25]. A recent study revealed that transducing the IGF-1 gene into chondrocytes contributed to improved cartilage repair with more intense staining for safranin-O and the maintenance of a three-layer structure as compared with the nontransduced control chondrocytes [26]. Another study demonstrated that IGF-1 promoted the chondrogenic differentiation capacity of bone marrow-derived MSCs, but in that study, IGF-1 also stimulated expression of a hypertrophic phenotype along with elevated osteogenic markers, suggesting the promotion of an osteogenic phenotype in this bone marrow-derived MSC population as well [27].

Since Syn-MSCs exhibit an inferior osteogenic differentiation capacity compared to bone marrow-derived MSCs [28, 29], the above discussed results could be source specific. To address this issue, it is of interest to assess the impact of enhanced IGF-1 expression on the biological phenotype of Syn-MSCs. In the present study, the IGF-1 gene was transferred into human Syn-MSCs and the influence of elevated expression of IGF-1 on their chondrogenic differentiation capacity was assessed.

## 2. Materials and Methods

### 2.1. Cell Culture

Human synovial-derived mesenchymal stem cells (hSyn-MSCs) were isolated and expanded as previously reported [30]. Briefly, synovium was obtained from 5 human donors (age = 17–35 years old; 2 females and 3 males) at the time of arthroscopic surgery of the knee (ex., ACL reconstruction, meniscus repair, or synovectomy). And they were free from any diagnosed diseases (Table 1). Before surgery, the patients received an explanation of the study, and consent was provided in accordance with a protocol approved by the Institutional Ethics Committee of Osaka University School of Medicine and affiliated hospital.

The synovial specimens were minced and digested with 0.2% collagenase (Collagenase-AOF Type A; Worthington Biochemical, Lakewood, NJ) in Dulbecco's modified Eagle's medium (DMEM; Gibco BRL, Life Technologies, Rockville, MD) for 2 hours in a shaking water bath at 37.0°C. The cells were collected by centrifugation and then cultured in a DMEM growth medium supplemented with 10% fetal bovine serum (FBS, Sigma-Aldrich, St. Louis, MO) and 1% penicillin-streptomycin (Sigma-Aldrich), with the medium replaced twice per week. These cells were defined as synovium-derived MSCs. When the cells had reached confluence, they were then washed with sterile phosphate-buffered saline (PBS), detached with trypsin-EDTA (0.25% trypsin + 1 mM EDTA; Gibco BRL, Life Technologies), and replated at a density of 3.0 × 10E3 cells/cm^2^. Cell passages were continued in the same manner when cells reached confluence. Cells at passages 3–5 were used in the present studies.

### 2.2. Construction of Lentiviral Vector

The plasmids used previously by Madry et al. [31] were initially provided for the current studies. The IGF-1 cDNA fragment was released from the plasmid and recombined with the SIN type lentiviral vector plasmid pLVSIN-IRES-ZsGreen1 (TAKARA, Shiga, Japan). The IGF-1-expressing lentiviral plasmid pLVSIN-IRES-IGF-1 ZsGreen and control plasmid pLVSIN-IRES-ZsGreeen1 stocks were generated by transfection of Lenti-X 293T cells (TAKARA). The Lenti-X HTX Packaging System (Clontech Laboratories, CA) was used. Lenti-X 293T cells were plated in DMEM with 10% dialyzed FBC (TAKARA) at a concentration of 1 × 10E6 cells per 100 mm diameter dish and then transfected the following day with 7 g of the plasmid. Supernatants of these transfected cells were collected on days 2 and 3 posttransfection and titrated using a Lenti-X-PCR Titration Kit (Clontech).

### 2.3. Lentiviral Vector

Syn-MSCs were cultured on retronectin (recombinant human fibronectin fragment (TAKARA BIO INC., Kusatsu, Shiga, Japan)) and coated on 6-well plates. The plates of cells and aliquots of the lentiviral stock were centrifuged at 1000 ×g for 2 hours at 32°C following retronectin-binding infection methods (MOI = 50). At 12 hours postviral infection, the medium was changed to the growth medium. After induction for 72 hours, the Syn-MSCs were subjected to triple lineage cell differentiation capability assays. The lentiviral-mediated gene induction in the cells was assessed by expression of mRNA transcript levels (RT-qPCR) and protein levels (Western blotting) for IGF-1.

### 2.4. Chondrogenic Differentiation

Chondrogenic differentiation of the MSCs was induced using a pellet-culture method. Briefly, the cells were cultured in polypropylene tubes at a density of 1 × 10E5 cells/tube in chondrogenic medium containing 1% insulin-transferrin-selenium supplement (ITS + Premix; Corning Inc., NY), 0.2 mM Asc-2P (Sigma-Aldrich), 50 ng/mL recombinant human BMP-2 (Osteopharma Inc., Osaka, Japan), and 2.5 ng/mL recombinant human TGF-beta3 (Peprotech, Rocky Hill, NJ). The chondrogenic medium was changed three times a week. To evaluate chondrogenic differentiation, the size of pellets was assessed, glycosaminoglycan (GAG) content determined, gene expression levels assessed, and histochemical assays performed. The size of pellets was measured using the transverse and longitudinal diameters of pellets under microscopic image processing software (Olympus Microscope, Nikon Image Plus, Japan) on days 7 and 14 after induction of differentiation. Glycosaminoglycan content was quantified using a Blyscan sulfated GAG assay kit (Biocolor Ltd., Carrick Fergus, UK). For histological and immunohistochemical assay, the pellets were fixed with 4% paraformaldehyde and embedding in paraffin. Approximately 5 *μ*m sections of the pellets were stained with safranin-O fast green or toluidine blue dye to detect sulfated GAGs. Proteinase K (DAKO, CA)-treated sections were probed with an anti-human collagen II (F-57, Kyowa Pharmachemical Co., Takaoka, Japan) or a collagen II antibody (eBioscience, San Diego, CA).

To examine gene expression levels in the pellets, total RNA was extracted with TRIzol (Life Technologies, MD), and complementary DNAs (cDNA) were synthesized with SuperScript VILO (Life Technologies). The mRNA expression levels for type II collagen alpha1 (Col2a1; Hs 00264051_m1) and Sox9 (Hs1001343_g1) genes were assessed by TaqMan real-time polymerase chain reaction (qRT-PCR, Applied Biosystems, Carlsbad, CA) using a StepOnePlus Real-Time PCR instrument (Applied Biosystems). The expression levels for type X collagen (Col10; Hs00166657_m1) and matrix metalloproteinase-13 (m-13; Hs00233992_m1) as hypertrophic chondrocyte markers and alkaline phosphatase (ALP; Hs03046558_s1) and Osterix (Hs01822874_m1) as osteogenic markers were evaluated by RT-qPCR. The values were normalized to glyceraldehyde-3-phosphate dehydrogenase (GAPDH; Hs02758891_g1) mRNA levels as a house keeping gene and an internal control.

### 2.5. Osteogenic and Adipogenic Differentiation

Osteogenic differentiation was induced when the cells were at approximately 80% confluency using a commercially osteogenic medium (StemPro Osteogenesis Differentiation Kit, Life Technologies GmbH, Darmstadt, Germany). The medium was changed three times a week. After one week of differentiation culture, the cells were evaluated for osteogenic differentiation by detection of ALP expression using an ALP staining method (BCIP/NBT Color Development Substrate, Promega). After three weeks, the cells were again evaluated for osteogenic differentiation using alizarin red staining (Muto Pure Chemicals Co., Tokyo, Japan). Quantitation of alizarin red staining was performed using the absorbance at 415 nm of the solution eluted from stained cells. Adipogenic differentiation induction of the cells was initiated when they had proliferated and were overconfluent using a commercially available adipogenic differentiation medium (StemPro Adipogenic Differentiation Kit, Life Technologies GmbH). After three weeks of differentiation, the cells were stained with oil red solution (0.5% Oil-Red, Sigma-Aldrich) to evaluate the lipid content of the cells as an indicator of adipogenic capability.

### 2.6. Statistical Analysis

Each in vitro experiment was repeated at least three times independently using different donor cell sources. The data were subjected to an independent samples *t*-test. The results are presented as mean ± standard deviation (SD) for triplicate determinations. For assessment of statistical difference, *p* values less than 0.05 were considered significant. All data analysis was performed using the statistical software “EZR” (Easy R) which is based on the R and R commander [32].

## 3. Results

### 3.1. Effect of IGF-1 Gene Transfer on Chondrogenic Differentiation

**Figure 1:**
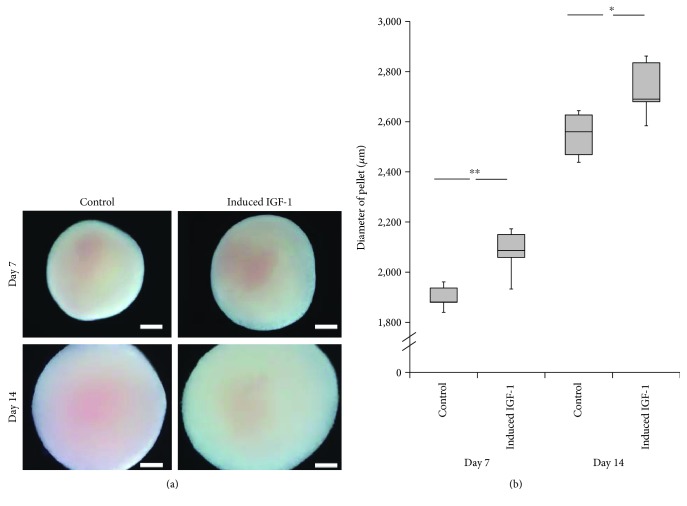
Diameters of the pellet cultures after 7 and 14 days of chondrogenic differentiation. (a) Microscopic findings: control (left) versus IGF-1 gene transferred cell-derived pellets (right). Showing results after 7 days (upper) and 14 days (lower). Scale bars = 500 *μ*m. (b) Graph showing the diameter of the pellet cultures after 7 and 14 days. Vertical axis: diameters (*μ*m); horizontal axis: the control (left) versus induced IGF-1 (right). Statistical analysis using independent samples *t*-test, ^∗∗^*p* < 0.01, ^∗^*p* < 0.05.

Previous research has demonstrated that the size of the pellet cultures is a good assessment parameter to evaluate achievement of chondrogenesis using pellet-culture differentiation methods [33]. Accordingly, we measured the diameters of the pellet developed from the pLVSIN-IGF-1-transfected MSCs. The mean diameters were 2080 ± 94.4 mm on day 7 and 2730 ± 116.0 mm on day 14. These values were significantly larger than those of the control group at 7 (1899 ± 47.8 mm (*p* < 0.01)) and 14 days (2548 ± 91.5 mm (*p* < 0.05)) (Figures 1(a) and 1(b)).

**Figure 2:**
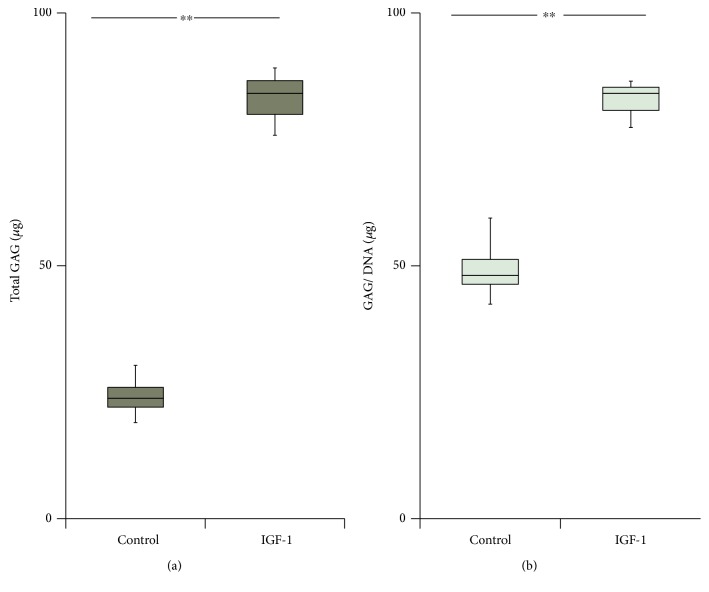
Gross and DNA-corrected glycosaminoglycan (GAG) content of pellet cultures (in *μ*g). The pellets were analyzed for GAG after 14 days in the differentiation medium using a Blyscan sulfated GAG assay kit. Control values (left) versus IGF-1 gene transfer values (right). (a) Box plot of gross GAG content of the pellet cultures. (b) Box plot of DNA-corrected GAG content. ^∗∗^*p* < 0.01.

Likewise, glycosaminoglycan (GAG) content was significantly higher by 3.6-fold (*p* < 0.01) in the IGF-1-induced cell pellets than in the control pellets at day 14 (Figure 2(a)). After normalizing to the DNA content, the GAG content in the IGF-1-transduced group was still significantly (1.7-fold) higher (*p* < 0.01) compared with values for the control group (Figure 2(b)).

**Figure 3:**
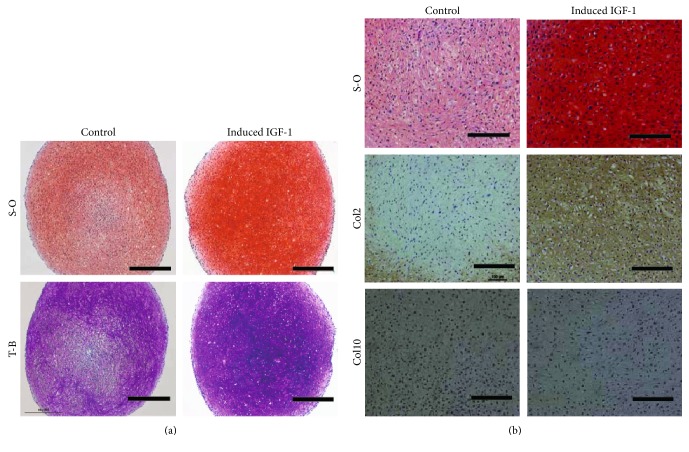
The results of histology and immunostaining assessments. (a) S-O; safranin-O (upper lane), T-B; toluidine blue (lower lane). Control (left) versus IGF-1 gene transferred cell-derived pellets (right). Scale bars = 500 *μ*m. (b) S-O; safranin-O (upper lane), Col2; type II collagen (middle), and Col10; type X collagen (lower) immunostaining. Control (left) versus IGF-1 gene transfer (right). Scale bars = 200 *μ*m.

Although all the pellets showed positive staining for safranin-O and toluidine blue regardless of IGF-1 introduction, the staining intensity was higher in samples from the IGF-1-transduced group than in those of the control group (Figure 3(a)). Thus, histology of the IGF-1-induced pellets exhibited more similarities to those of normal, hyaline cartilage. Notably, there were no apparent increases in the presence of hypertrophic chondrocytes with large and round shapes in the IGF-1 transduced pellets as compared with the control pellets (Figure 3(b) upper lane). In other donor samples, similar results were obtained (Supplementary Figure 2A, 2B available online at https://doi.org/10.1155/2017/5804147).

The results of immunostaining revealed (Figure 3(b)) more intense staining for type II collagen within the IGF-1-transduced pellets than in the control pellets. Conversely, staining for type X collagen, a marker for the hypertrophic phenotype was negligible in both groups, suggesting the presence of hypertrophic chondrocytes with in the pellets of both groups was minimal (Figure 3(b)).

**Figure 4:**
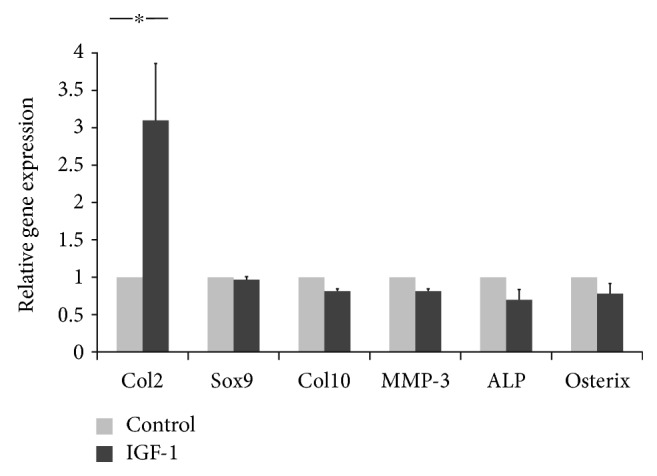
Specific gene expression levels in MSCs under chondrogenic conditions. Gene expression analyses of human synovial MSCs overexpressing IGF-1 after induction of chondrogenic differentiation were performed using real-time RT-PCR. The MSCs were transduced with a pLVSIN-IRES-ZsGreen1 (control; left) or the pLVSIN-IRES-IGF-1 ZsGreen vector (IGF-1; right) and cultured in a chondrogenic differentiation medium for 14 days. The values for each gene were normalized to the GAPDH house keeping gene levels used as the internal control. Ct values were generated for each target gene. ^∗^Statistically significant compared with control values (*p* < 0.05). GAPDH: glyceraldehyde-3-phosphate dehydrogenase. Col2 (type II collagen alpha1) and Sox9 were assessed as chondrogenic markers. Col10 (type X collagen) and MMP-13 (matrix metalloproteinase 13) were assessed as hypertrophic chondrocyte markers. ALP (alkaline phosphatase) and Osterix were assessed as osteogenic markers.

We also assessed RNA from IGF-1-transduced cells and control cells for mRNA transcript levels for the chondrogenic marker gene type II collagen alpha1 (Col2A1), as well as the hypertrophic marker type X collagen (Col10), matrix metalloproteinase 13 (MMP-13), and the osteogenic marker genes ALP and Osterix. Expression levels for the chondrogenic marker gene Col2A1 were significantly higher (3.1-fold) in the IGF-1-transduced group compared with those in the control group (*p* < 0.05). In contrast, mRNA expression levels for the hypertrophic chondrocyte marker genes (ColX and MMP-13), and the osteogenic marker genes (ALP and Osterix) were not significantly higher in the pellets from the IGF-1-transduced cells than those from the control pellets (Figure 4).

### 3.2. Osteogenic and Adipogenic Differentiation of IGF-1-Transduced Syn-MSCs

**Figure 5:**
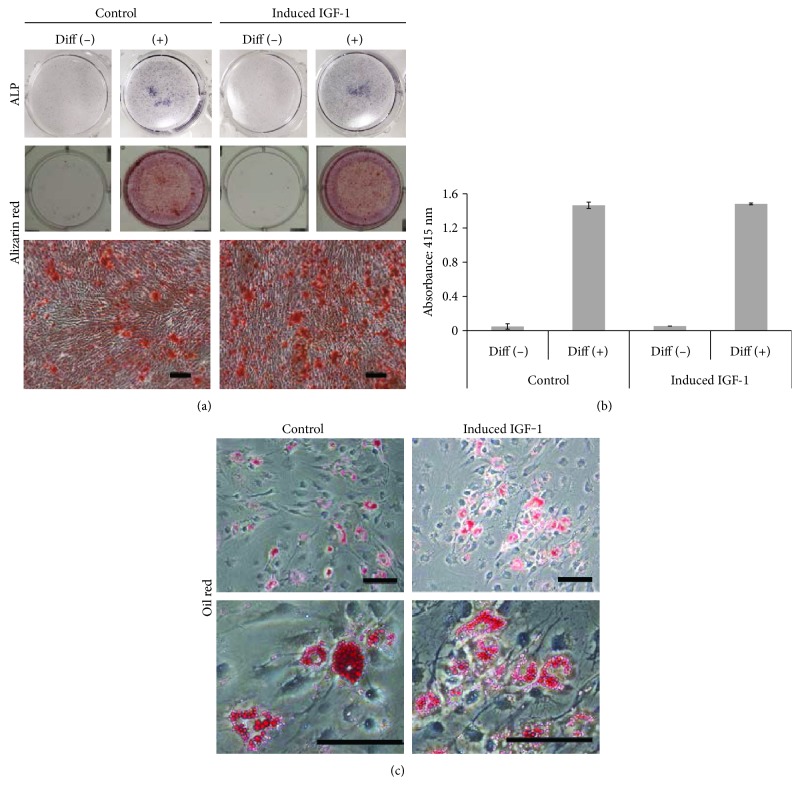
Effect of IGF-1 transduction on osteogenic and adipogenic differentiation of human Syn-MSC. (a) Osteogenic differentiation assay using ALP staining after 1 week of differentiation (upper). Alizarin red staining after 3 weeks of differentiation (lower). Scale bars = 500 *μ*m. (b) Quantitated alizarin red staining values after osteogenic differentiation in the control versus induced IGF-1 groups. (c) Adipogenic differentiation after 3 weeks in a differentiation medium using oil red staining. Scale bars = 100 *μ*m.

The effect of IGF-1 introduction into Syn-MSCs on osteogenic and adipogenic lineage differentiation capacity was also investigated. There were no detectable differences in ALP staining at 7 days between the IGF-1 group samples and those for the control group (Figure 5(a)). After 21 days of osteogenic differentiation, both groups exhibited similar staining with alizarin red (Figure 5(a)), and quantitated values for both groups were not significantly different (Figure 5(b)).

After 21 days of exposure to adipogenic differentiation culture conditions, detection of lipid droplets associated with adipogenic differentiation was observed to be similar in both groups (Figure 5(c)).

The above described results are a representative of similar findings obtained with three of the donors of Syn-MSCs. Other donors were used to confirm only parts of the complete characterization. The control group, which was transduced with the pLVSIN-IRES-ZsGreen1 plasmid lacking the IGF-1 insert was used for cell proliferation assays and found to not differ from the properties of nontransduced cells (Supplementary Figure 3). Therefore, the nontransduced cells were defined as the control group for all subsequent assessments.

## 4. Discussion

The present study has demonstrated that lentiviral vector-mediated IGF-1 gene transfer specifically enhances the chondrogenic differentiation capacity of human Syn-MSCs and does so without any detectable impact on osteogenesis and adipogenesis differentiation of these cells.

The therapeutic use of MSCs for cartilage repair has been advocated to overcome the limitations of autologous chondrocyte implantation approaches, such as issues related to the dedifferentiation of chondrocytes during expansion culturing and the impact of removal of undamaged cartilage from the same joint. In their review, Filardo et al. [9] indicated that one-half of the 60 clinical trials which were using MSCs for cartilage repair were reported in the last 3 years. However, it has also been revealed that MSC-based therapies have some limitations regarding the quality of the repair tissue due to the contamination with fibrocartilaginous tissue [22]. Such incompletely regenerated tissue was shown to have inferior biomechanical properties as compared with normal cartilage [22]. Therefore, it is necessary to improve the repair quality of MSC-based therapies in order to move towards tissue regeneration. A number of approaches have been investigated for this purpose. Along with the alternative use of pluripotent stem cells such as embryonic stem cells or induced pluripotent stem (iPS) cells as the cell source [34–39], manipulation of the biological phenotype of MSC could be a one potential direction out of several that include inclusion exertion of low oxygen tension during cell culture [40], application of exogenous growth factors or cytokines [41–43], and gene transfer of specific target molecules [44, 45].

We hypothesized that IGF-1 gene transfer could improve the chondrogenic capacity of human Syn-MSCs, analogous to the study by Madry et al. [26], where the IGF-1 gene was introduced into bovine chondrocytes. To enhance the efficiency of IGF-1 gene introduction, we utilized an established lentiviral vector system. Recently, the SIN type lentiviral vector has become one of the most popular tools for gene transfer to mammalian cell based on its efficiency, safety, and convenience [46]. Transfer of the IGF-1 gene transfer into MSCs was confirmed at the levels of mRNA transcripts and protein expression (Supplementary Figure 1A, 1B), and it occurred without detectably altering the cell surface marker expression profile (Supplementary Figure 1C) or the differentiation capacity of the cells towards the osteogenic and adipogenic lineages. In contrast, introduction of the IGF-1 gene promoted chondrogenic differentiation of Syn-MSCs to a tissue phenotype which was morphologically more similar to hyaline cartilage. It should also be pointed out that in the present study, IGF-1 gene transfer to Syn-MSCs promoted chondrogenesis without detectable enhancement of the hypertrophic or osteogenic phenotype. Frisch et al. reported that expression of osteogenic and hypertrophic marker genes was increased by overexpression of IGF-1 in bone marrow-derived MSCs, possibly related to the propensity of bone marrow-derived MSCs to differentiate towards the osteogenic lineage along with some chondrogenesis [27, 45]. Although the differences observed between the two studies may have been affected by differing conditions for gene transfer and cell culture, it is also likely that the action of IGF-1 on the differentiation characteristics of Syn-MSCs versus bone marrow-derived MSCs may be also be quite different. In support of that conclusion, Goodrich et al. reported ectopic bone formation within repair tissue following the implantation of bone marrow-derived MSCs into chondral defects [47]. Such findings suggest that potential osteogenic differentiation of MSCs can occur in vivo during cartilage repair. Along with the previous report that premature induction of hypertrophy during in vitro chondrogenesis of MSCs correlates with calcification after ectopic implantation [48], stimulation of the osteogenic differentiation capacity of MSCs should be avoided when being used for cartilage repair. In this regard, exposure to IGF-1 during bone marrow-derived MSC-based cartilage repair could increase the risk of unfavorable bone formation and thus is not recommended. Conversely, IGF-1-transduced Syn-MSCs appear to exhibit lineage-specific enhanced chondrogenic capacity, and the clinical implication of these observations is that such cells could be a promising approach for manipulating cartilage repair towards more complete tissue regeneration.

One of limitations of this study is that although we identified a unique phenotypic change towards an enhanced chondrogenic differentiation capacity of human Syn-MSCs via IGF-1 gene transfer, we could not assess whether the differences are dependent on donor ages and sex. We used cell sources that were available, but these were young donors (17–35 years old) and the numbers were limited. However, we have previously investigated the detailed chondrogenic capacities of a number of human Syn-MSCs of differing ages, and we did not detect any obvious differences in the phenotype of individual Syn-MSCs assessed [49]. It does however remain controversial whether human Syn-MSCs populations exhibit different chondrogenic capacities dependent on age or sex. In addition, we did not assess the biomechanical characterization of the cartilage tissues or the prototypical stability of the cartilaginous tissues in vivo after implantation. Further studies in these areas are needed for detailed clarification of the potential of the IGF-1-transduced MSC to foster cartilage regeneration.

## 5. Conclusion

Lentivirus-mediated IGF-1 gene transfer to human Syn-MSC promotes the chondrogenic differentiation capacity of the cells without stimulating either the hypertrophic or osteogenic phenotype, and thus, may have enhanced potential for cartilage repair.

## Supplementary Material

Supplemental Information. Supplemental Figure 1: The leniviral-mediated gene intduction of IGF-1 into the human Syn-MSC. A: showing mRNA transcript levels by RT-PCR using human IGF-1 specific primer pairs. The control (left) vs. IGF-1 gene transferred cell (right). B: Protein expression by Western blotting with anti-IGF1 antibody (ab9572, Abcam, Cambridge, UK). The control (left) vs. IGF-1 gene transferred cell (right). C: Cell surface marker expression of Control and IGF-1 transferred cells by fluorescence-activated cell sorting (FACS) analysis. Supplemental Figure 2: The results of histology assessments from the other cases. The control (left) vs. IGF-1 gene transferred cell derived pellets (right). T-B; Toluidine-Blue (upper lane),S-O; Safranin-O (lower lane). Left: Scale bars =500μm, right: Scale bars =200 μm.

## Figures and Tables

**Table 1:** Donor information of synovial mesenchymal stem cells.

Patient no.	Age	Gender	Surgery	Comorbid diagnosis
1	17	Male	Synovectomy	None
2	22	Male	ACLR	None
3	29	Female	ACLR	None
4	33	Male	ACLR + MR	None
5	35	Female	ACLR	None

ACRL: anterior cruciate ligament reconstruction; MR: meniscal repair.

## Abbreviations

GAG: Glycosaminoglycan
M.O.I.: Multiplicity of infection
MSCs: Mesenchymal stem cells.
