# Supplementary material for: IGF-1 Gene Transfer to Human Synovial MSCs Promotes Their Chondrogenic Differentiation Potential without Induction of the Hypertrophic Phenotype
Source: Stem Cells Int. 2017 Jun 27;2017:5804147. doi: 10.1155/2017/5804147 (PMC5504993; doi:10.1155/2017/5804147)

Supplemental Information

Supplemental Figure1 : The leniviral-mediated gene intduction of IGF-1 into the human Syn-MSC

A: showing mRNA transcript levels by RT-PCR using human IGF-1 specific primer pairs.

The control (left) vs. IGF-1 gene transferred cell (right).

B: Protein expression by Western blotting with anti-IGF1 antibody (ab9572, Abcam, Cambridge, UK).

The control (left) vs. IGF-1 gene transferred cell (right).

C: Cell surface marker expression of Control and IGF-1 transferred cells by fluorescence-activated cell sorting (FACS) analysis.

Supplemental Figure2 : The results of histology assessments from the other cases

The control (left) vs. IGF-1 gene transferred cell derived pellets (right).

T-B; Toluidine-Blue (upper lane), S-O; Safranin-O (lower lane)

Left: Scale bars =500μm, right: Scale bars =200 μm

Supplemental figure1A, B

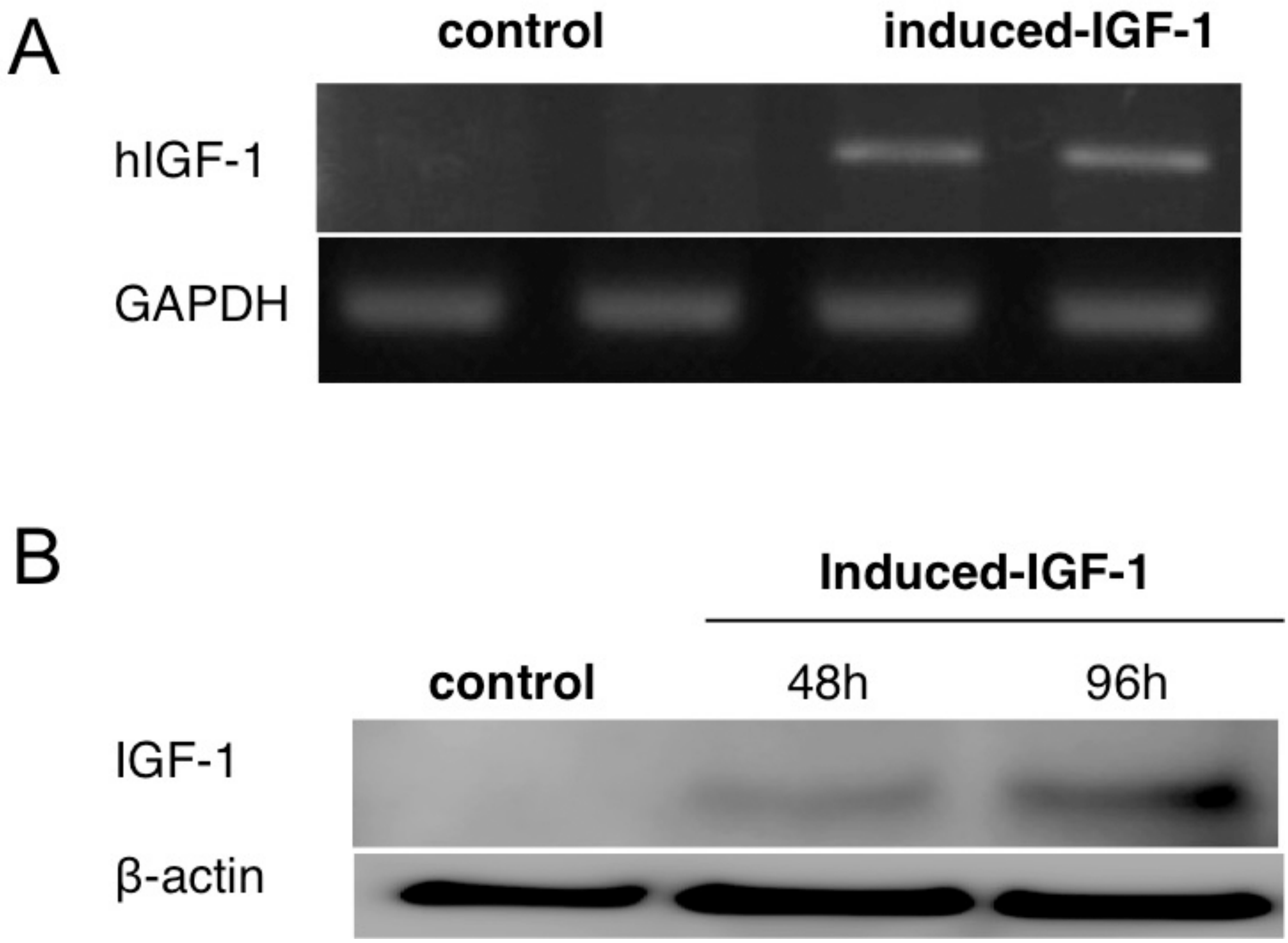

Supplemental figure1C

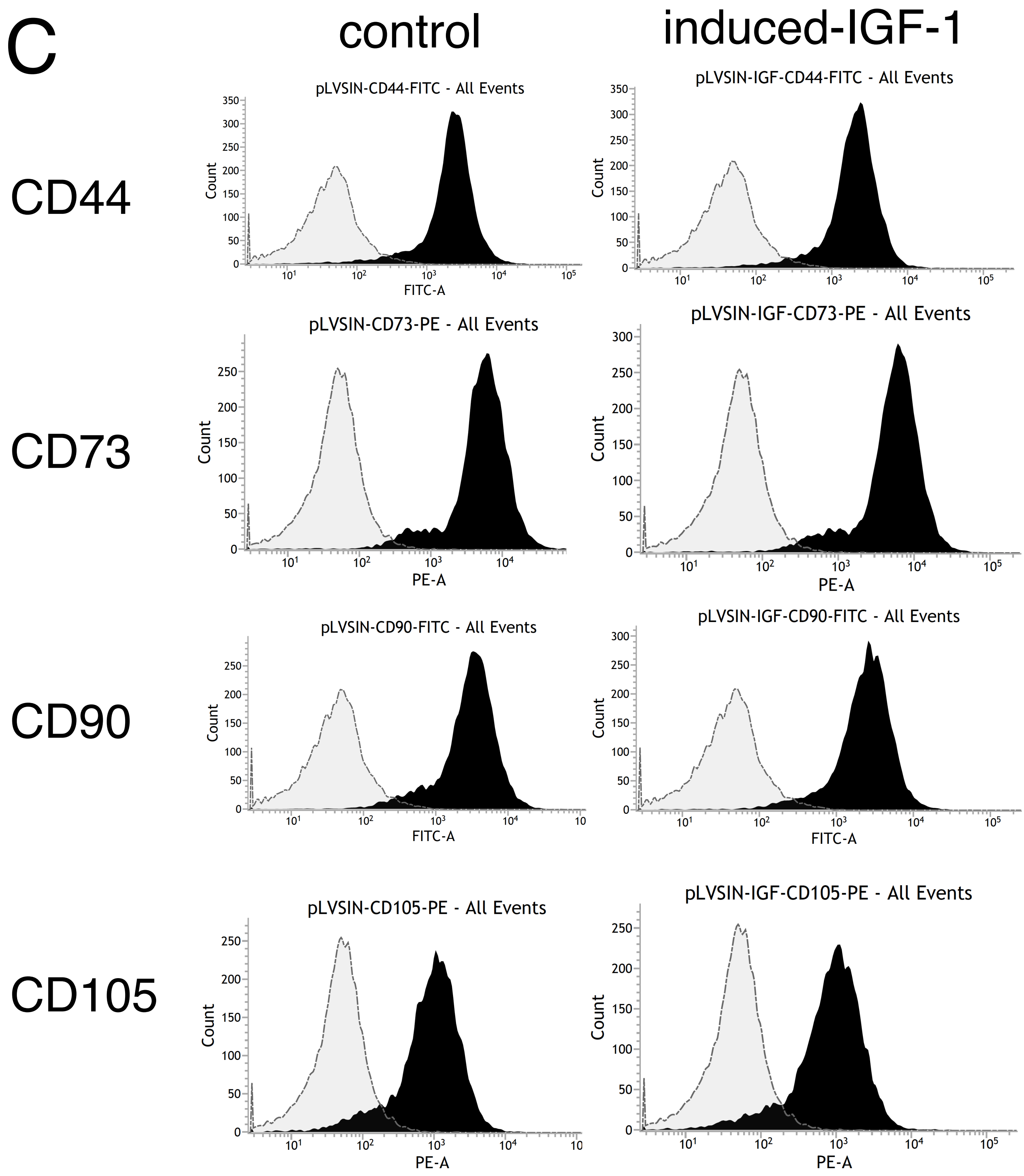

Supplemental figure 2

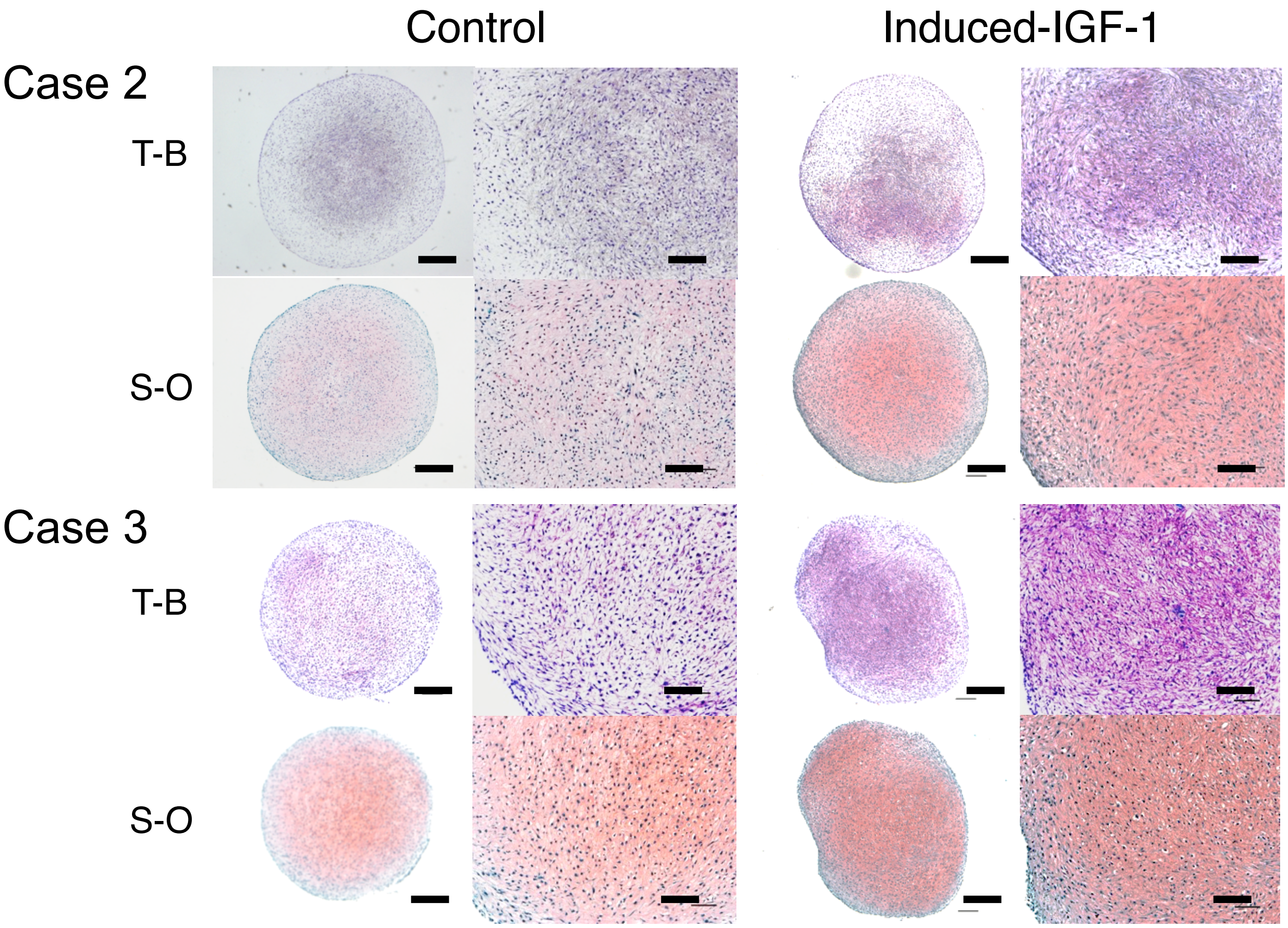

Supplement: Supplementary file 1 — Supplemental Information. Supplemental Figure 1: The leniviral-mediated gene intduction of IGF-1 into the human Syn-MSC. A: showing mRNA transcript levels by RT-PCR using human IGF-1 specific primer pairs. The control (left) vs. IGF-1 gene transferred cell (right). B: Protein expression by Western blotting with anti-IGF1 antibody (ab9572, Abcam, Cambridge, UK). The control (left) vs. IGF-1 gene transferred cell (right). C: Cell surface marker expression of Control and IGF-1 transferred cells by fluorescence-activated cell sorting (FACS) analysis. Supplemental Figure 2: The results of histology assessments from the other cases. The control (left) vs. IGF-1 gene transferred cell derived pellets (right). T-B; Toluidine-Blue (upper lane),S-O; Safranin-O (lower lane). Left: Scale bars =500μm, right: Scale bars =200 μm. [file 5804147.f1.pdf]
