# Supplementary figures and images for: IGF-1 Gene Transfer to Human Synovial MSCs Promotes Their Chondrogenic Differentiation Potential without Induction of the Hypertrophic Phenotype
Source: Stem Cells Int. 2017 Jun 27;2017:5804147. doi: 10.1155/2017/5804147 (PMC5504993; doi:10.1155/2017/5804147)

## Slide 1
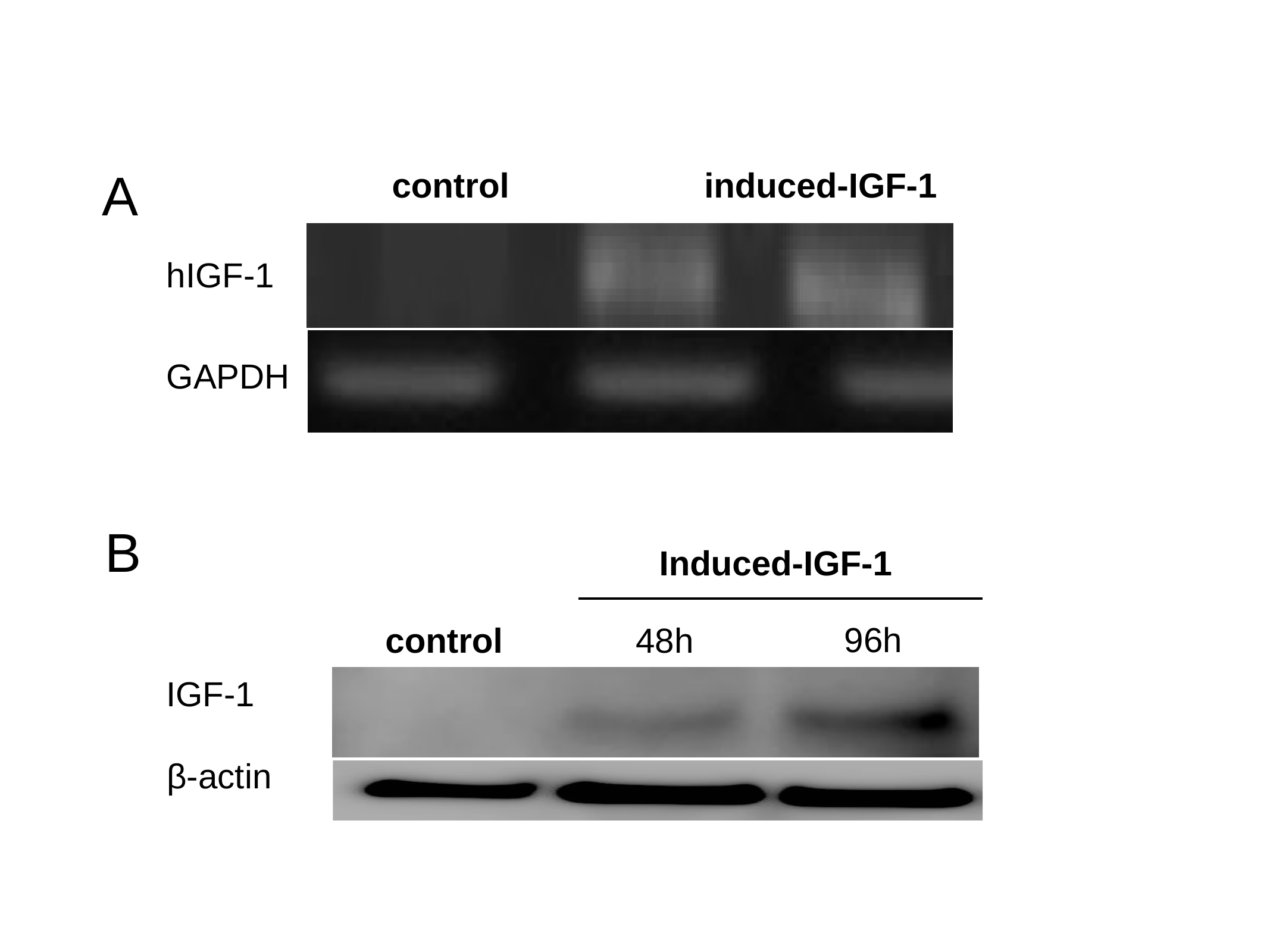

A
control
induced-IGF-1
hIGF-1
GAPDH
B
Induced-IGF-1
96h
control
48h
IGF-1
β-actin

Supplement: Supplementary file 2 [file 5804147.f2.pptx]

## Slide 1
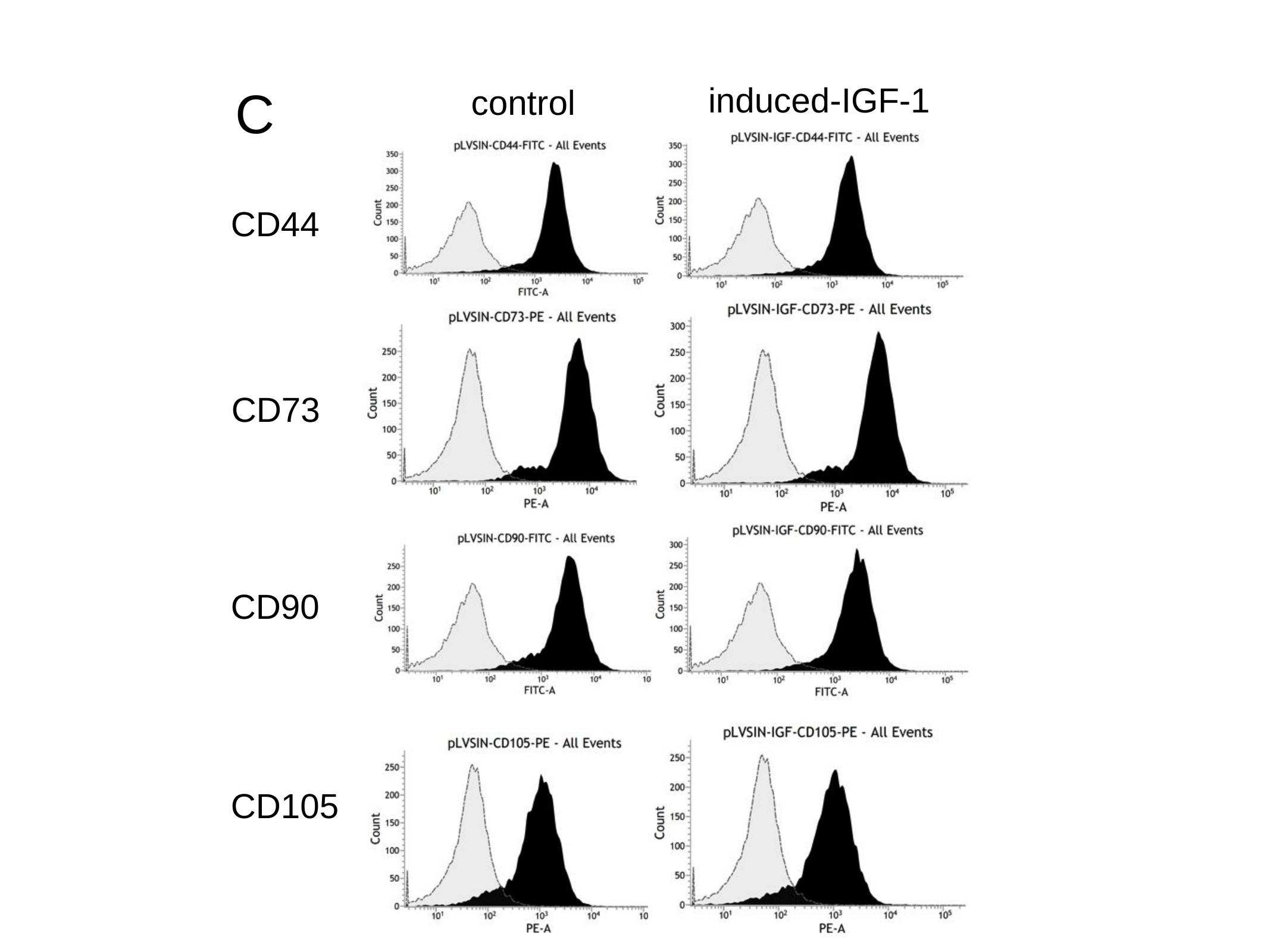

induced-IGF-1
control
C
CD44
CD73
CD90
CD105

Supplement: Supplementary file 3 [file 5804147.f3.pptx]

## Slide 1
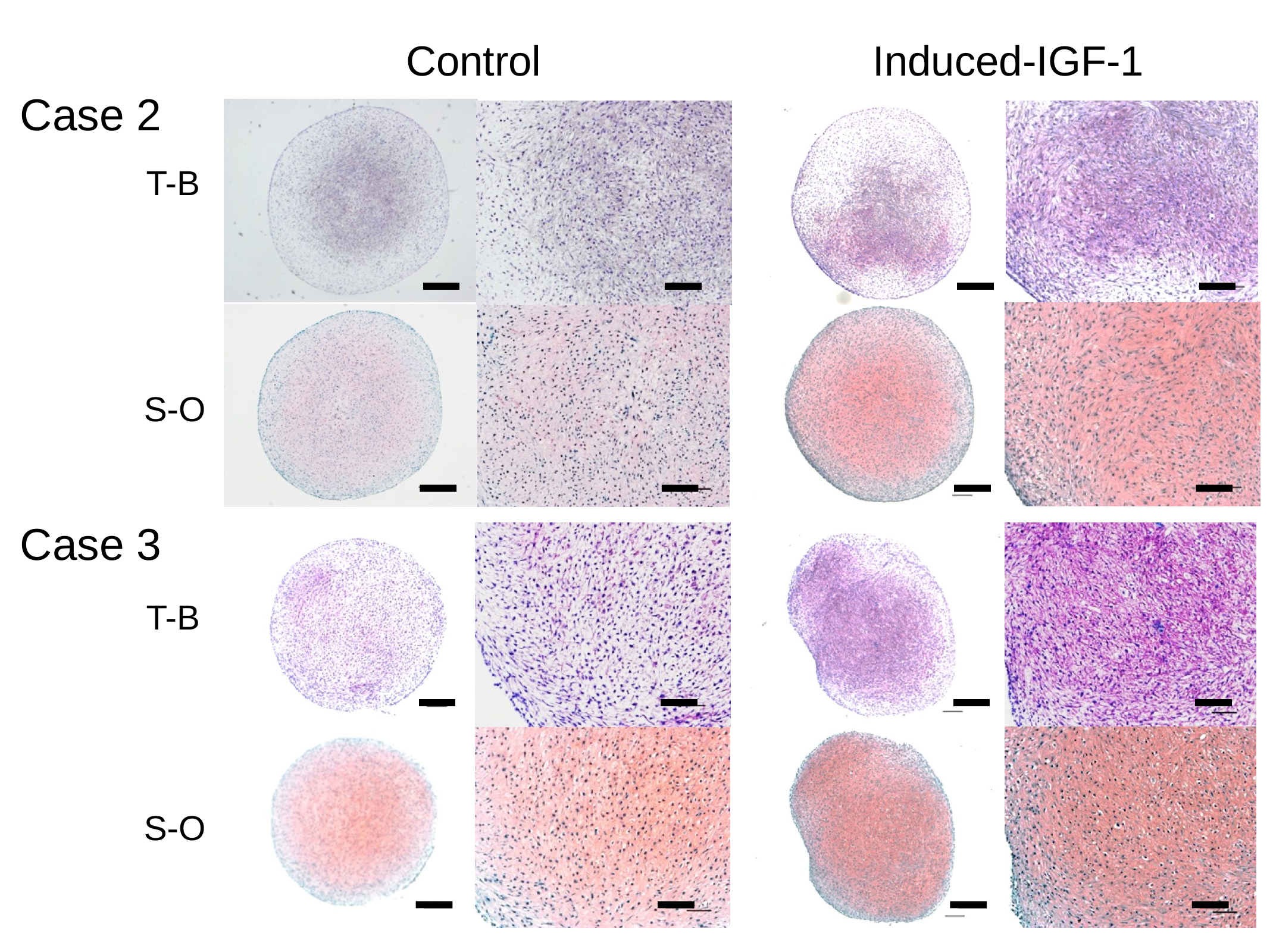

Control
Induced-IGF-1
Case 2
T-B
S-O
Case 3
T-B
S-O

Supplement: Supplementary file 4 [file 5804147.f4.pptx]

## Slide 1
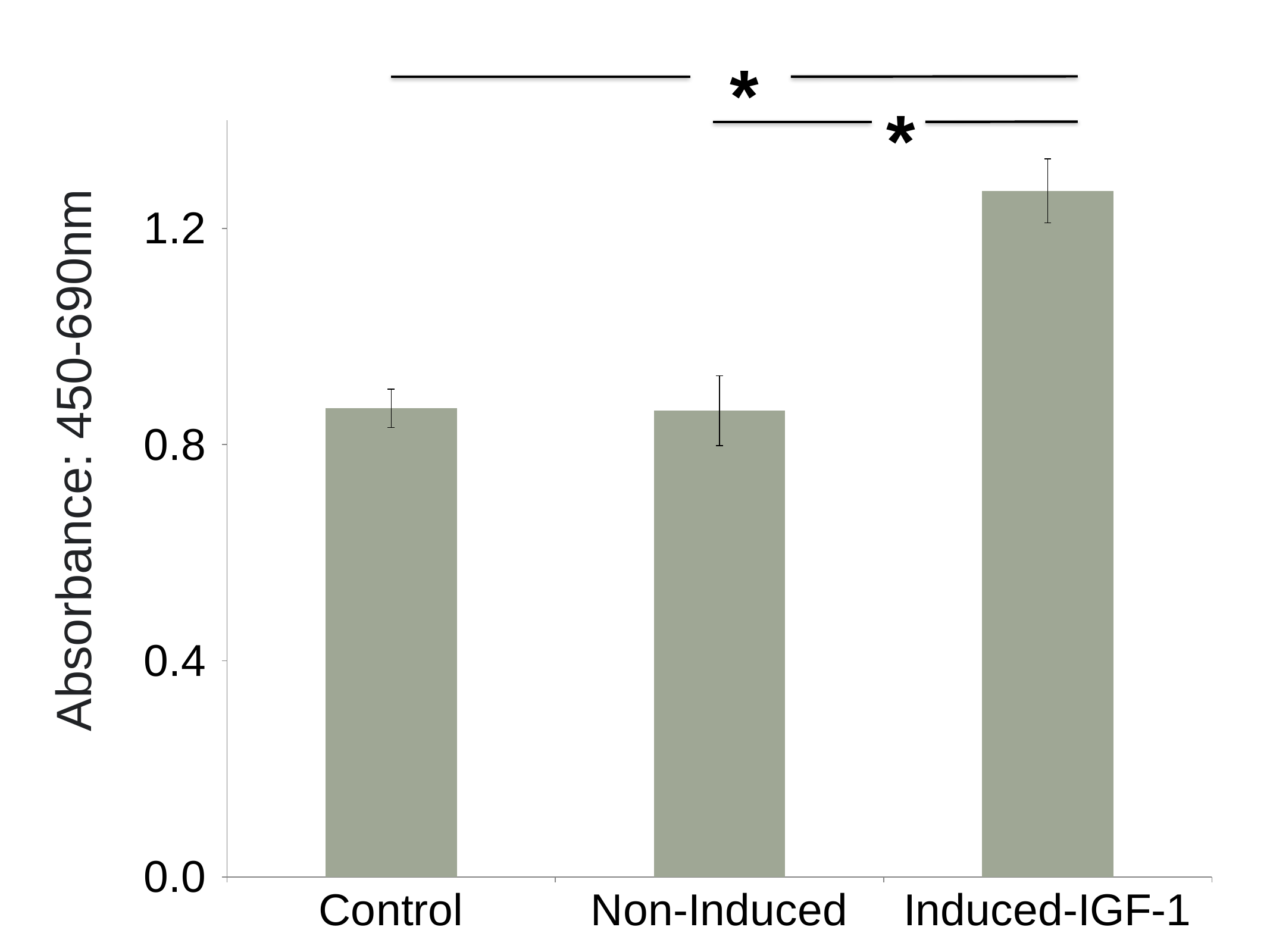

*
### Chart
| Category |
|---|*
### Chart
| Category | 1.0x10E4 450-690nm |
|---|---|
| Control | 0.8672 |
| Non-Induced | 0.862666666666667 |
| Induced-IGF-1 | 1.269566666666667 |Absorbance: 450-690nm

Supplement: Supplementary file 5 [file 5804147.f5.pptx]
